# SEGN: Inferring real-time gene networks mediating phenotypic plasticity

**DOI:** 10.1016/j.csbj.2020.08.029

**Published:** 2020-09-05

**Authors:** Libo Jiang, Christopher H. Griffin, Rongling Wu

**Affiliations:** a Beijing Advanced Innovation Center for Tree Breeding by Molecular Design, Beijing Forestry University, Beijing 100083, China; b Center for Computational Biology, College of Biological Sciences and Technology, Beijing Forestry University, Beijing 100083, China; c Applied Research Laboratory, The Pennsylvania State University, University Park, PA 16802, USA; d Center for Statistical Genetics, Departments of Public Health Sciences and Statistics, The Pennsylvania State University, Hershey, PA 17033, USA

**Keywords:** Gene regulatory networks, Dynamic transcriptional plasticity, Phenotypic plasticity, Euphrates poplar, Salt stress

## Abstract

The capacity of an organism to alter its phenotype in response to environmental perturbations changes over developmental time and is a process determined by multiple genes that are co-expressed in intricate but organized networks. Characterizing the spatiotemporal change of such gene networks can offer insight into the genomic signatures underlying organismic adaptation, but it represents a major methodological challenge. Here, we integrate the holistic view of systems biology and the interactive notion of evolutionary game theory to reconstruct so-called systems evolutionary game networks (SEGN) that can autonomously detect, track, and visualize environment-induced gene networks along the time axis. The SEGN overcomes the limitations of traditional approaches by inferring context-specific networks, encapsulating bidirectional, signed, and weighted gene-gene interactions into fully informative networks, and monitoring the process of how networks topologically alter across environmental and developmental cues. Based on the design principle of SEGN, we perform a transcriptional plasticity study by culturing Euphrates poplar, a tree that can grow in the saline desert, in saline-free and saline-stress conditions. SEGN characterize previously unknown gene co-regulation that modulates the time trajectories of the trees’ response to salt stress. As a marriage of multiple disciplines, SEGN shows its potential to interpret gene interdependence, predict how transcriptional co-regulation responds to various regimes, and provides a hint for exploring the mass, energetic, or signal basis that drives various types of gene interactions.

## 1 Introduction

Phenotypic plasticity is the capacity by which an organism changes its phenotypes in direct response to environmental change [1]. By producing phenotypic novelties that can better adapt to novel and stressful environments, phenotypic plasticity, through genetic assimilation, facilitates evolutionary change and speciation [2], [3], [4], [5], [6]. The pattern of how phenotypic traits respond to environmental variation is thought to be mediated by genes [7], [8], [9], [10], [11], but previous studies have been mostly focused on single differentially-expressed genes [12], [13], [14]. Such reductionist thinking is a powerful approach for identifying key genes, but may be insufficient to characterize the comprehensive genomic signature of phenotypic plasticity, because mounting evidence shows that adaptation to novel environments require the change of myriad genes that interact and work together to form intricate but coordinated networks [15], [16], [17], [18]. Spurred by the recent development of high-throughput sequencing techniques, there has been a surge of interest inferring gene regulatory networks for particular cellular processes [19], [20], but the real-time identification of a comprehensive relationship between these networks and phenotypic plasticity has proven to be challenging.

In this study, we develop a computational model that can reconstruct time-varying networks of gene interactions and track real-time alterations of network architecture causing phenotypic plasticity. Most existing approaches can only reconstruct a single (context-agnostic) network from expression data, failing to characterize causal networks from transcriptional plasticity to phenotypic plasticity. We view gene interactions as a game, in which each gene (i.e., player) tends to maximize its expression and impact both based on its own optimal strategy and its accrued knowledge of the environment affected by other genes. The application of game theory to gene expression analysis has been explored in previous studies, showing its formidable capacity to disentangle genomic complexities [21], [22], [23]. We integrate *evolutionary game theory* [24] to formulate the time-varying expression level of each gene in terms of its intrinsic capacity and extrinsic influence by other genes through a mixed ordinary differential equation (ODE). The implementation of functional clustering [25], [26], [27] and variable selection [28] facilitates the *systematic* construction of high-dimensional ODE from genome-wide expressed genes. In the end, we can encapsulate all possible interactions into systems evolutionary game networks (SEGN) that quantify how each gene interacts with every other gene as a regulatory mechanism that guides the organisms’ response to environmental and developmental signals.

Because of their dynamic property, SEGN can unravel and track real-time alterations of network architecture during biological processes. Based on the design principle of SEGN, we design and conduct a genomic experiment with saline-varying treatments using clonal replicates of Euphrates poplar (*Populus euphratica*), the only woody tree that can survive in the saline desert [29], [30], [31]. We reconstruct SEGN for the phenotypic plasticity of salt resistant-related physiological traits. SEGN characterize some previously unknown gene co-regulation patterns that are responsible for the tree’s tolerance and resistance to salts.

## 2 Model overview

### 2.1 Defining dynamic phenotypic and transcriptional plasticity

We initiate a genomic experiment using cloneable plants that allow the same individual to be replicated genotypically. The same genotype of a plant species was grown under two contrast treatments each with multiple replicates, aimed to study the genetic mechanisms of how the organism responds to environmental change. We measure a series of phenotypic traits of interest and importance to evolutionary and breeding studies. In practice, the time schedule of measurement may be unevenly spaced; for example, data are usually measured more densely at the early stage than at later stages of the experiment. Let (*Z*_1_*_k_*(*t*_1_), ….…, *Z_pk_*(*t_T_*)) denote the phenotypic values of *p* traits measured at time *t* (*t* = *t*_0_, *t*_1_, …, *t_T_*), respectively, under treatment *k* (*k* = 1, 2). Note that *t*_0_ can be used as a start time point for both treatments. We also measure the time-dependent expression levels of *m* transcriptomic genes, following the same time schedule as used for phenotypic monitoring. We use (*Y*_1_*_k_*(*t*_1_), ..., *Y_mk_*(*t_T_*)) to denote the expression values of *m* genes at time *t* under treatment *k*.

To quantify phenotypic plasticity, we take differences of trait values under two treatments [11]. Thus, the dynamic phenotypic plasticity (DPP) of trait *j* (*j* = 1, …, *n*) is defined as

(1a) $$z_{i}\left(t\right)=Z_{j2}\left(t\right)-Z_{j1}\left(t\right)$$

(1b) $$=u_{j}\left(t\right)+\varepsilon_{j}\left(t\right),$$

where *u_j_*(*t*) is the time-varying expectation of phenotypic plasticity for trait *j* and *ε_j_*(*t*) is the residual error. We hypothesize that treatment-induced alteration of transcriptional profiles is a force that drives the organism to change its trait value from one treatment to the other. The first step of testing this hypothesis is to calculate the transcriptional plasticity of expression dynamics which is defined as the difference of expression amounts between two treatments over time. Thus, the dynamic transcriptional plasticity (DTP) of arbitrary gene *i* (*i* = 1, …, *m*) at time *t* is calculated as

(2a) $$y_{i}\left(t\right)=Y_{i2}\left(t\right)-Y_{i1}\left(t\right)$$

(2b) $$=g_{i}\left(t\right)+e_{i}\left(t\right),$$

where the DTP calculated from Eq. (2A) is partitioned into its expected value (*g_i_*(*t*)) and residual error (*e_i_*(*t*)), which describe how the expression of gene *i* changes from treatment 1–2 over developmental time as a result of the expectation and the random error, respectively. A traditional line of thinking is to test how DPP is statistically dependent on DTP by regressing *z_j_*(*t*) on *y_i_*(*t*) using standard least-square analysis approaches. Yet, our aim is beyond this; we seek to reconstruct the regulatory network of *m* genes and formulat a model to casually link this network to the corresponding phenotypic network.

### 2.2 Integrating evolutionary game theory into gene networks

Genes, co-inhabiting a nucleus, often regulate each other to form a complex interaction network. Such a network behaves like an ecological community in which one species may compete for access to resources or cooperate symbiotically with other species to drive community dynamics. How a gene chooses a cooperative (activation) or competitive (inhibition) strategy can be explained by game theory. Game theory, originated in economic research [32], models the payoff of one player based on the strategy implemented by the other player. The application of game theory has been largely popularized by the concept of the Nash equilibrium, a proposed solution of a non-cooperative game, at which each rational agent tends to choose an optimal strategy to maximize its payoff, conditioned on the strategies of its opponents, as long as the latter remains unchanged [33]. By combining game theory and evolutionary biology, Smith and Price [24] formulated evolutionary game theory to interpret how frequency dependent fitness drives strategies to evolution [34]. This theory’s core is the concept of an evolutionarily stable strategy regarded as an equilibrium refinement of the Nash equilibrium and its extension to population evolution. However, Smith and Price’s evolutionary game theory serves as the *static* analysis tool of evolutionary stability because it does not attempt to model how strategies change in a population. By adding the time dimension, we expand evolutionary game theory to its *dynamic* domain, making it possible to explicitly model the change of strategy frequencies in the population. Such a dynamic evolutionary game theory does not need to define a notion of evolutionary stability. Instead, by specifying a population dynamic model, all of the standard stability concepts from dynamical systems can be used.

Dynamic evolutionary game theory proposes a mathematical model for specifying how a gene is expressed differently over time through its own strategy and the strategies implemented by other genes. In other words, such a model can decompose the overall expression of a gene into its underlying independent expression component (determined by its intrinsic capacity) and dependent expression component (determined by its extrinsic influence). By considering all *m* genes, we develop an *m*-dimensional system of ODE to model the dynamic change of gene expression, expressed as

(3) $$\frac{dg_{i}}{\mathrm{dt}}=Q_{i}\left(g_{i}\left(t:\Theta_{i}\right)\right)+\sum_{{i^{\text{'}}=1,i}^{\text{'}}\neq i}^{m}Q_{ii^{\text{'}}}\left(g_{i^{\text{'}}}\left(t:\Theta_{ii^{\text{'}}}\right)\right),i=1,\cdots ,m$$

where the change of DTP for each gene *i* (*i* = 1, …, *m*) per unit time is split into two components: the *independent* expression (the first term), which occurs when the focal gene *i* is assumed to be in isolation and is specified by a gene-specific smoothing function *Q_i_*(*g_i_*(*t*;Θ*_i_*)); and the *dependent* expression (the second term), which reflects the aggregated effect of all other genes *i*′ (*i*′ = 1, …, *i*-1, *i* + 1,…, *m*) on the focal gene and is specified by the sum of smoothing functions $\sum_{{i^{\text{'}}=1,i}^{\text{'}}\neq i}^{m}Q_{ii^{\text{'}}}\left(g_{i^{\text{'}}}\left(t:\Theta_{ii^{\text{'}}}\right)\right)$. Here, Θ*_i_* and Θ*_ii_*_′_ are a set of ODE parameters that describe the independent DTP of a gene and how the DTP of the focal gene depends jointly on other genes, respectively. Let *P_i_*(*t*) and $P_{ii^{\text{'}}}\left(t\right)$denote the integrals of independent component *Q_i_*(*g_i_*(*t*;Θ*_i_*)) and dependent component $Q_{ii^{\text{'}}}\left(g_{i^{\text{'}}}\left(t:\Theta_{ii^{\text{'}}}\right)\right)$, respectively. Then, we code *P_i_*(*t*) as a node and $P_{ii^{\text{'}}}\left(t\right)$ as an edge into an *m*-dimensional network.

Equation (3) provides a general ODE framework for inferring systems evolutionary game networks (SEGNs). To reconstruct large-scale, omnidirectional, and omnigenic gene-gene interactions, we need to develop powerful statistical algorithms for solving the ODEs in Eq. (3). In the Supplementary Text, we describe an algorithmic procedure for estimating the ODE parameters under a maximum likelihood setting.

### 2.3 Biological interpretation of SEGNs

The SEGN is a fully informative network constructed from bidirectional, signed, and weighted gene interactions. The pattern of how gene *i* is affected by gene *i*′ can be assessed by $P_{ii^{\text{'}}}\left(t\right)$. If this value is positive, zero, or negative, then this suggests that gene *i*′ activates, is neutral to, or inhibits gene *i*, respectively. By comparing $P_{ii^{\text{'}}}\left(t\right)$ and $P_{i^{\text{'}}i}\left(t\right)$, we can classify all gene interactions into five qualitatively different types:

• *Synergism* by which two interactive genes activate each other. This can be seen if both $P_{ii^{\text{'}}}\left(t\right)$ and $P_{i^{\text{'}}i}\left(t\right)$ are positive;
• Antagonism by which two interactive genes inhibit each other. This can be seen if both $P_{ii^{\text{'}}}\left(t\right)$ and $P_{i^{\text{'}}i}\left(t\right)$ are negative;
• *Directional synergism* by which gene *i*′ activates gene *i* but the latter is neutral to the former. This can be seen if $P_{ii^{\text{'}}}\left(t\right)$ is positive but $P_{i^{\text{'}}i}\left(t\right)$ is zero;
• *Directional antagonism* by which gene *i*′ inhibits gene *i* but the latter is neutral to the former. This can be seen if $P_{ii^{\text{'}}}\left(t\right)$ is negative but $P_{i^{\text{'}}i}\left(t\right)$ is zero;
• *Altruism/exploitation* in which one gene activates the other but the latter inhibits the former. If $P_{ii^{\text{'}}}\left(t\right)$ is positive whereas $P_{i^{\text{'}}i}\left(t\right)$ is negative, this suggests that gene *i*′ offers altruism to gene *i*, or say, gene *i* exploits gene *i*′.

It is possible that the two genes may peacefully coexist when they do not affect each other. This can be seen if both $P_{ii^{\text{'}}}\left(t\right)$ and $P_{i^{\text{'}}i}\left(t\right)$ are zero. The SEGN is also a quantitative network, because each activation or inhibition is quantified by a value. If $P_{ii^{\text{'}}}\left(t\right)$ and $P_{i^{\text{'}}i}\left(t\right)$ are positive and their values are equal, the synergism of two genes *i* and *i*′ is regarded as symmetric synergism. If $P_{ii^{\text{'}}}\left(t\right)$ and $P_{i^{\text{'}}i}\left(t\right)$ are positive but their values are not equal, then synergism becomes asymmetrical synergism. Similarly, we can distinguish between symmetric antagonism and asymmetrical antagonism. Table 1 condenses the important features of the SEGNs. Taken together, the definitions and interpretations of various patterns of gene co-regulation can facilitate the exploration of the mass, energetic, or signal basis for each interaction.

**Table 1:** Qualitative definition of gene interaction and its quantitative characterization by the SEGN model.

		Quantitative description		
No	Qualitative definition	$P_{ii^{\text{'}}}\left(t\right)$		$P_{i^{\text{'}}i}\left(t\right)$
1	Symmetric synergism	+	=	+
2	Asymmetric synergism	+	≠	+
3	Directional synergism toward *i*	+	>	0
4	Directional synergism toward *i*′	0	<	+
5	Altruism toward *i* or exploitation by *i*	+		−
6	Altruism toward *i*′ or exploitation by *i*′	−		+
7	Symmetric antagonism	−	=	−
8	Asymmetric antagonism	−	≠	−
9	Directional antagonism toward *i*	−	0	
10	Directional antagonism toward *i*′	0		−
11	Coexistence	0		0

Note: ${P_{ii^{\text{'}}}\left(t\right)andP}_{i^{\text{'}}i}\left(t\right)$ are the dependent expression levels of gene *i* by gene *i*′ and gene *i*′ by gene *i*, respectively.

The central themes of network reconstruction include sparsity, stability and causality [35]. As described above, the implementation of ODEs meets the causality property of a network by determining the direction of gene interaction. As shown in the Supplementary Text, the statistical procedure for learning the SEGN is formulated under the maximum likelihood and convex optimality setting. Thus, we think of the various strategies used by each gene as it interacts with different genes as leading to achieve maximum stability of the interaction network. Modularity theory asserts that biological entities are often specified for different functions and, therefore, are organized into distinct modules within which entities are more functionally correlated with each other than with those from other modules [36]. This theory allows us to cluster a large number of genes into functionally different modules by implementing the functional clustering algorithm [25], [26], [27]. As predicted by network theory, there is a limit to the number of links owned by each node in a network [37]. We can implement variable selection methods to detect the number of the most significant genes that affect a focal gene. Taken together, we can reconstruct high-dimensional, multiscale and sparse networks.

Networks are regarded as snapshots of biological systems at different times. Uncovering the dynamic nature of transcriptional networks can shed light on the genomic mechanisms that drive phenotypic plasticity. As a function of time *t*, $P_{ii^{\text{'}}}\left(t\right)$ can be calculated at any time point from *t* = 0 to *T* and, therefore, establishes a real-time visualization of gene networks during biological processes.

### 2.4 Hierarchic networks linking transcriptional plasticity to phenotypic plasticity

To test whether and how gene networks determine the DPP of physiological traits that are associated with salt resistance, we construct a set of ODE-based regression models as follows:

(4) $$\frac{du_{j}}{\mathrm{dt}}=R_{j}\left(u_{j}\left(t:\Omega_{j}\right)\right)+\sum_{j^{\text{'}}=1,j^{\text{'}}\neq j}^{n}R_{jj^{\text{'}}}\left(u_{j^{\text{'}}}\left(t:\Omega_{jj^{\text{'}}}\right)\right)+\sum_{i=1}^{m}S_{\mathrm{ji}}\left(g_{i}\left(t:\Psi_{\mathrm{ji}}\right)\right),j=1,\cdots ,n$$

where the change of DPP of trait *j* (*j* = 1, …, *n*) per unit time is decomposed into three components: the independent DPP of the trait (assuming no interaction with other traits and genes) specified by function R*j* (*u*(t: Ω*j*)), the accumulated dependent DPP affected by other traits specified by the sum of functions $\sum_{j^{\text{'}}=1,j^{\text{'}}\neq j}^{n}R_{jj^{\text{'}}}\left(u_{j^{\text{'}}}\left(t:\Theta_{jj^{\text{'}}}\right)\right)$, and the accumulated dependent DPP regulated by genes specified by the sum functions $\sum_{i=1}^{m}S_{\mathrm{ji}}\left(g_{i}\left(t:\Theta_{\mathrm{ji}}\right)\right)$. Functions containing parameters Ω*j*, Ω*_jj’_*, and $\Psi j_{i}$ can be fitted by nonparametric approaches, such as B-spline or Legendre orthogonal polynomials.

We integrate a system of trait and gene-mixed ODEs in Eq. (4) and a system of purely gene-based ODEs in Eq. (3) to form an expanded system of ODEs that can model causal gene-trait relationships. Variable selection approaches are implemented for this expended system to determine a subset of the most significant predictors (including traits and genes). We then chart three networks; the gene network, the trait network, and the gene-trait causal network. The third network establishes a bridge that links the gene network to the trait network.

## 3 Results

To validate the biological relevance of our SEGN model, we carried out a genomic experiment by culturing Euphrates poplar clones in salt-stress and salt-free conditions (see the Supplementary Text). We measured six salt-responsive physiological traits, i.e., superoxide dismutase (SOD), malonaldehyde (MDA), catalase (CAT), peroxidase (POD), soluble sugar content, and protein content, and a total of 1819 salt-responsive genes from poplar roots before treatment and at four different time points after treatment (Table S1).

### 3.1 Functional clustering

**Fig. 1:**
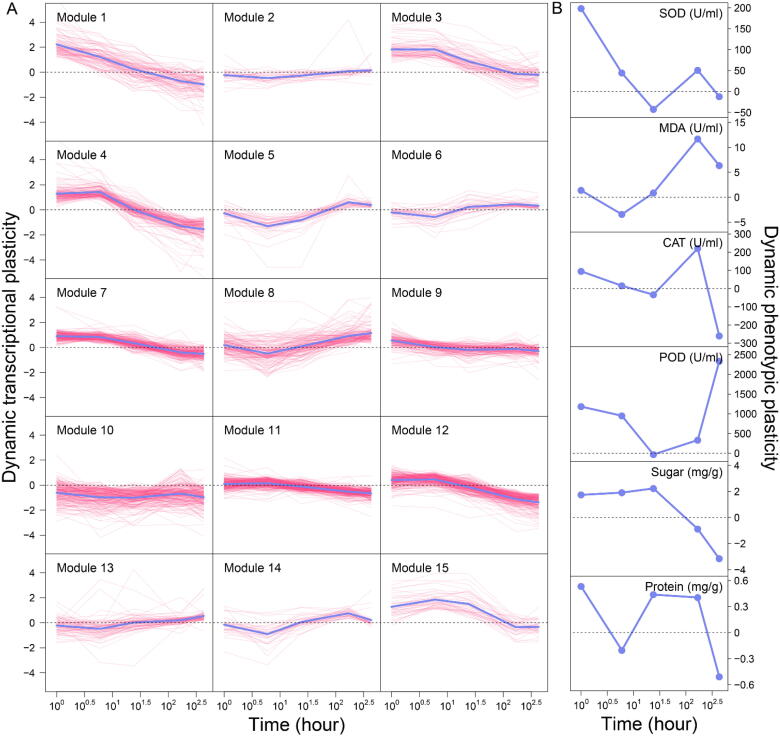
Dynamic transcriptional plasticity (DTP) of 15 distinct modules among 1,819 salt-responsive genes (A) and dynamic phenotypic plasticity (DPP) of six salt resistance-related physiological traits (B) measured from roots of Euphrates poplar clones grown under salt-free and salt-stress conditions. Purple thin lines are the DTP curves of individual genes within a module, and thick blue lines represent the mean curves of all genes from this module. (For interpretation of the references to colour in this figure legend, the reader is referred to the web version of this article.)

According to Eqs. (1a), (2a), we calculated the DTP of each gene and the DPP of each trait. The DTP and DPP quantify the degree of how a gene or trait responds to salt condition, respectively. We found that the genes studied display distinct dynamic patterns of responsiveness to the salt treatment. We used Jiang et al.’s [27] Skellam clustering approach to categorize the 1819 genes into 15 modules (Fig. 1A). This is an optimal number of modules according to AIC. Table 2 gives the number of genes detected within each module. The 15 modules are different in terms of the amount and direction of DTP and its rate of change. Several modules, such as 1, 3, 7, 10, 11, and 12, decrease their DTP consistently with time, although the rate of decrease varies among these modules. The DTP of a few modules, like 2, consistently increases with time. Many modules, including 4, 5, 6, 8, 9, 13, 14, and 15, change their DTP periodically with time, with the sharpest change occurring in the early stage of salt treatment. We found that all six physiological traits are highly plastic to salinity, although their DDP display different patterns (Fig. 1B). Overall, week 2 after treatment is a turnover point at which ing is a turning point at which almost all traits respond to salt stress differently from their previous pattern of response.

**Table 2:** DTP-based functional modules of 1819 differentiated genes coded each decomposed to its independent component and dependent component including passive regulation and active regulation by other modules.

Module	#Gene	Passive Regulation	Active Regulation
**1**	98	8^+^(0.84), 9^+^(0.83), 13^–^(0.72)	3^+^(0.28), 11^+^(0.82)
**2**	30	4^+^(0.62), 7^+^(0.16), 13^+^(0.77)	9^+^(0.82)
**3**	73	1^+^(0.29), 4^-^(0.66), 9^+^(0.50)	
4*	141	9^+^(0.91), 15^+^(0.86)	2^+^(0.62), 3^–^(0.66), 5^–^(0.69), 7^+^(0.81), 9^+^(0.88), 10^+^(0.80), 11^+^(0.07), 12^–^(0.38), 14^–^(0.13)
**5**	41	4^–^(0.69), 10^–^(0.13)	10^+^(0.93), 15^+^(0.77)
**6**	39	9^+^(0.81), 10^+^(0.66)	
**7**	220	4^+^(0.81), 10^–^(0.42)	2^+^(0.16), 8^+^(0.55)
**8**	145	7^+^(0.55), 9^+^(0.79)	1^–^(0.72)
9*	146	2^+^(0.82), 4^+^(0.88), 10^+^(0.76)	1^+^(0.83), 3^+^(0.50), 4^+^(0.91), 6^+^(0.81), 8^+^(0.79), 13^+^(0.40), 14^–^(0.01)
10*	219	4^+^(0.80), 5^+^(0.93)	5^-^(0.69), 6^+^(0.66), 7^–^(0.42), 9^+^(0.76), 13^–^(0.84), 14^+^(0.98), 15^+^(0.21)
**11**	264	1^+^(0.82), 13^+^(0.51)	
**12**	246	4^–^(0.38),, 13^–^(0.63)	
**13**	79	9^+^(0.40), 10^–^(0.84)	1^–^(0.72), 2^+^(0.77), 12^–^(0.63)
**14**	36	4^–^(0.13), 9^–^(0.01), 10^+^(0.98)	
**15**	42	5^+^(0.93), 10^+^(0.21)	4^+^(0.86)

Note: Column 2 contains the number of genes within each module. Column 3 are the modules by which a focal module is regulated through activation (+) or inhibition (–). Column 4 are the modules that a focal module actively regulates through activation (+) or inhibition (–). Hub modules are indicated by an asterisk. Numbers in brackets are correlation coefficients between two modules across time points.

### 3.2 How genes interact dynamically in response to salt stress

We used the ODEs of Eq. (3) to draw the mean DTP curve of each module, which is partitioned into its independent and dependent expression components. The magnitude and pattern of dependent DTP expression curves reflect the dynamic relationships of a specific focal module with other individual modules. We find that all modules are regulated by other modules over time although there is considerable variability in the frequency and strength of regulation among modules. Table 2 provides detailed information about the pattern of co-regulation among all 15 modules. Co-regulation includes passive regulation by which a focal module is activated or inhibited by other modules and active regulation by which a focal module activates or inhibits other modules. According to network theory, those modules that display more regulation than the average are defined as hubs. It is important to define the hubs because they play a dominant role in mediating network structure and behavior [38].

**Fig. 2:**
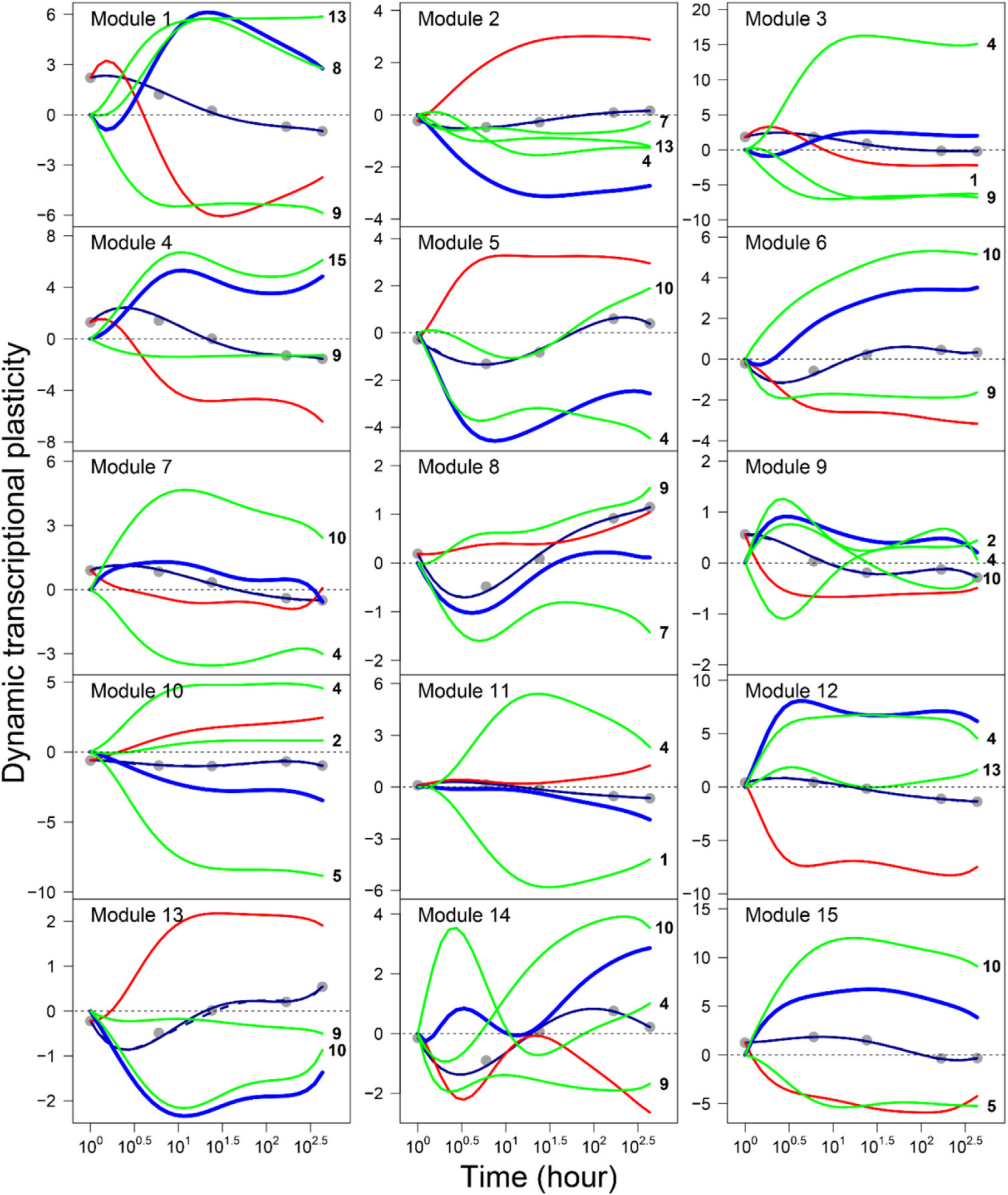
Mean dynamic transcriptional plasticity (DTP) curve of all genes within each module (black solid line) fitted by a nonparametric Legendre orthogonal polynomial (LOP) approach, which is dissected into its independent DTP curve (red line) and dependent DTP curve regulated by other labeled modules (green line). The thick blue line is the summed dependent DTP of all interacting modules. (For interpretation of the references to colour in this figure legend, the reader is referred to the web version of this article.)

The co-regulation among different modules can be illustrated graphically (Fig. 2). For example, the DTP of module 4 decreases gradually with time after a short increase in the early stage of response to salt stress, but the independent DTP of this module displays a much greater rate of time-dependent decrease. This difference results from the accumulated positive effect of the extremely strong positive dependent DTP triggered by module 15 and a slight negative dependent DTP from module 9. In this sense, module 4 performs its biological function, largely relying on co-regulation mainly by module 15 and secondly by module 9. While it is regulated by the two modules, module 4 actively regulates many other modules, making it one of the leaders or hubs among the 15 modules. Yet, module 4 chooses different strategies to interact with other modules. It activates the expression of modules 3, 10, 11, and 12 increasingly with time, but inhibits the expression of module 5 over time. Interestingly, module 4 activates modules 9 and 14 in one stage but inhibits them in an other stage. We found that the strength and dynamic change of regulation by module 4 varies considerably, depending on which module it regulates.

By comparing the dependent DTP curve of module 4 affected by module 9 and the dependent DTP curve of module 9 affected by module 4, we found that module 4 is altruistic toward module 9 at the early stage of salt stress during which the former activates the latter but the latter inhibits the former. However, the strength of this relationship decreases strikingly with time because module 4 decreases its activation after trees start to sense salt stress. In the late stage, module 4 still activates module 9, but module 9 does not affect module 4, suggesting that they establish a directional synergism relationship. As can be seen from the above analysis, the pattern of gene-gene interaction may change with time, which can be detected and quantified by our method.

### 3.3 How a coarse-grained gene network drives DPP

We reconstruct the SEGN of transcriptional plasticity at coarse- and fine-grained levels. Genes from different modules display distinct patterns of expression plasticity, whereas those from the same module respond to saline stress in a broadly similar pattern. Thus, the network inferred from DTP values of different modules helps to explain the coarse-grained relationship of gene regulation through modularity, and a fine-grained view of regulation can be gained from the networks describing the DTP of individual genes within the same module. Gene networks at different levels are used as a predictor of the DPP of salt-responsive traits.

**Fig. 3:**
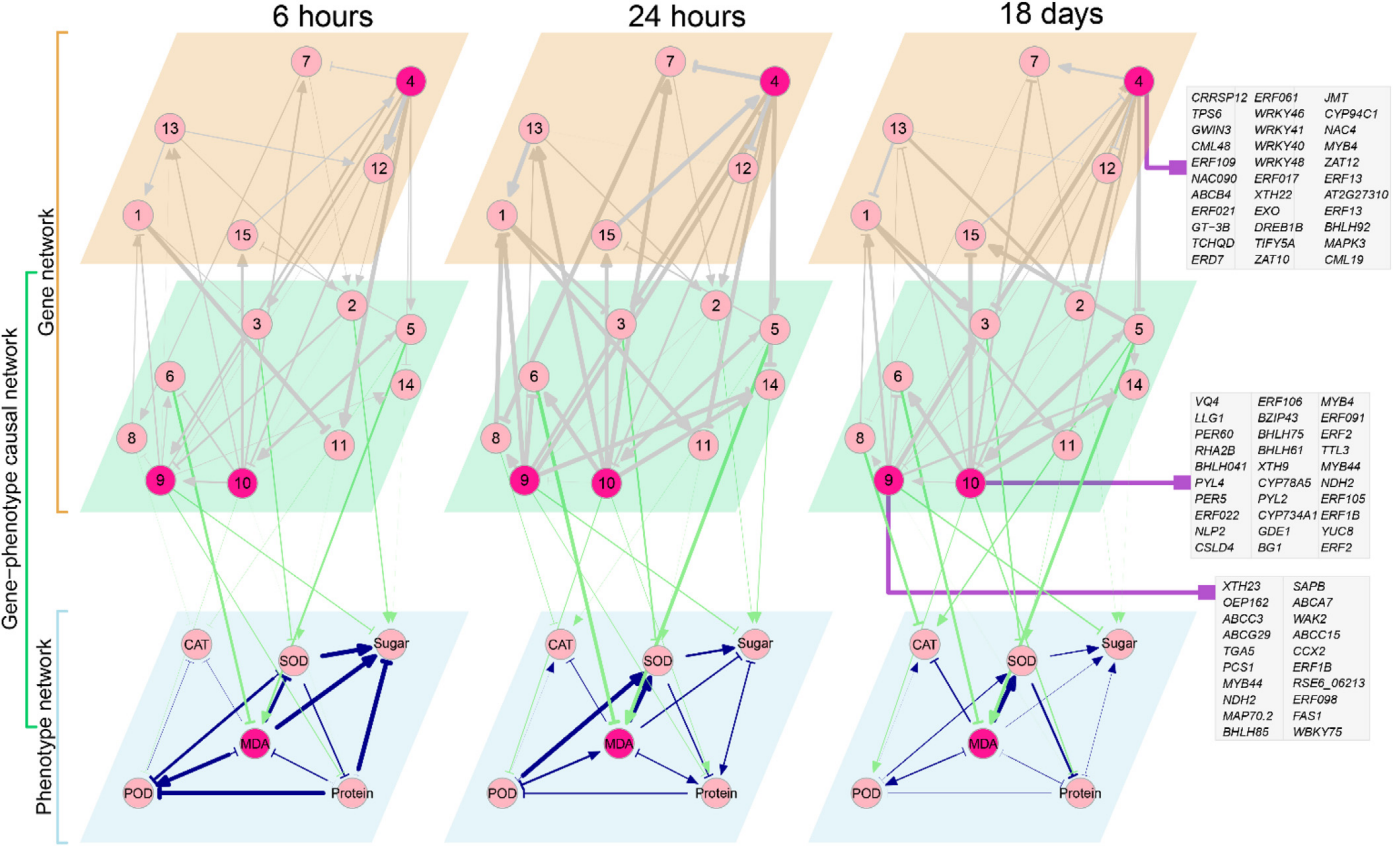
The transcriptional network of 15 modules and its link to the phenotypic network of six physiological traits through a causal network. Nodes are genes or traits indicated by circles (where dark circles denote the hubs of networks), and edges are gene regulation, activation or inhibition, indicated by arrowed line and T-shaped line, respectively. The thickness of lines are proportional to the strength of gene regulation. Hub modules 4, 9, and 10 are composed of many genes (whose names are shown in grey squares) with known biological functions. Some pairs of modules, like 4 and 9, are mutually regulated.

We build a system of trait-based ODE to reconstruct a phenotypic network, and regressed the phenotypic network on the gene network to obtain a gene-phenotype causal network at any time point from *t* = 0 to *T.* These networks are the snapshots of Euphrates poplar’s tolerance to salt stress, representing fully informative graphs in terms of interaction direction, sign, and size. Fig. 3 illustrates these real-time networks reconstructed at 6 h, 24 h, and 18 days after salt treatment. The six physiological traits form stable networks, but change structurally through time. MDA is a hub trait that plays a dominant role in modulating the network by affecting all other traits, but its impact varies dramatically with time. For example, MDA for sugar activates in the early stage but shifts to inhibition in the middle stage, and returns to activation in the late stage. From the phenotypic networks, one can visualize how each trait links dynamically with other traits.

Such time-varying phenotypic networks may arise from the dynamic change of gene module networks overwhelmed by directional synergism and directional antagonism. Of 15 gene modules, nine display direct links to the traits, forming a gene-phenotype causal network (Fig. 3). We found that each trait is regulated directly by at least one gene module and indirectly by many other modules that interact with the former. In the transcriptional networks reconstructed at 6 h, 24 h, and 18 days after salt treatment, modules 4, 9, and 10 are consistently hubs that each regulate more than seven modules through activation or inhibition. Modules 9 and 10 directly promote the increase of protein content, and they also pleiotropically promote sugar and POD, respectively, which are linked indirectly through protein content. Module 4 is not directly linked with traits, but it affects the phenotypic network through numerous pathways composed of genes in the trait-phenotype causal network. The three hub modules each contain genes that can be related to known saline resistant-related biological and molecular processes.

Module 4 contains 136 genes, of which as many as 22 are the transcriptional factors including *ERF109*, *AIL6*, *ERF017*, *ERF020*, *AIL5*, *MYB4*, *GT3B*, *BHLH92*, *WRKY48*, *WRKY40*, *WRKY41*, and *WRKY46* [39]. It has been well known that *ERF109*, *AIL6*, *ERF017* and *ERF020,* belonging to the AP2/ERF transcriptional family are believed to govern plants’ response to a variety of adverse stressors by participating in the signal transduction of salicylic acid, jasmonic acid, ethylene and abscisic acid [40]. Other genes *WRKY48*, *WRKY40*, *WRKY41*, and *WRKY46* are attributed to transcriptional family, WRKY, a family known to regulate the reaction of plants to adverse stressors [41]. Module 4 activates many modules, including 5, 10, 14, 2, 7, 9, 3, 13, and 12, but is activated by 15 and 9, suggesting that module 4 plays a leadership role in mediating network behavior.

Module 9 contains genes that mostly regulate the function of molecule transportation, such as potassium ion transmembrane transporter activity and primary active transmembrane transporter activity [42]. It is interesting to see that module 9 not only regulates module 1 directly, but also does so indirectly via many paths, such as path 1 by which module 9 regulates 4, 4 regulates 10, 10 regulates 13, and 13 regulates 1, path 2 by which module 9 regulates 4, 4 regulates 5, 5 regulates 10, 10 regulates 7, 7 regulates 8, 8 regulate 1; and path 3 by which module 9 regulate 8, and 8 regulate 1 (Fig. 3). Module 1 contains a number of genes related to many salt-responsive biological processes, such as the response the jasmonic acid mediated signaling pathway, the oxidation-reduction process, the cellular response to jasmonic acid stimulus, and the cellular response to oxygen-containing compound [42]. Module 10 also contains many genes related to biological processes (Fig. 3). Taken together, the three hub modules 4, 9, and 10 may be major drivers that control the regulatory process of salt resistance in distinct ways.

Although the structure of the modular network shares some similarities at three stages of salt response, some remarkable discrepancy exists along time axis (Fig. 3). For example, module 9 triggers directional antagonism toward module 1, with the degree increasing from early to middle stages of salt response. But this directional antagonism shifts to directional synergism when salt response enters a late stage. By contrast, module 10 activates module 15 at both the early and middle stage, but the former exerts directional antagonism toward the latter in the last stage.

### 3.4 How fine-grained gene networks drive DPP

**Fig. 4:**
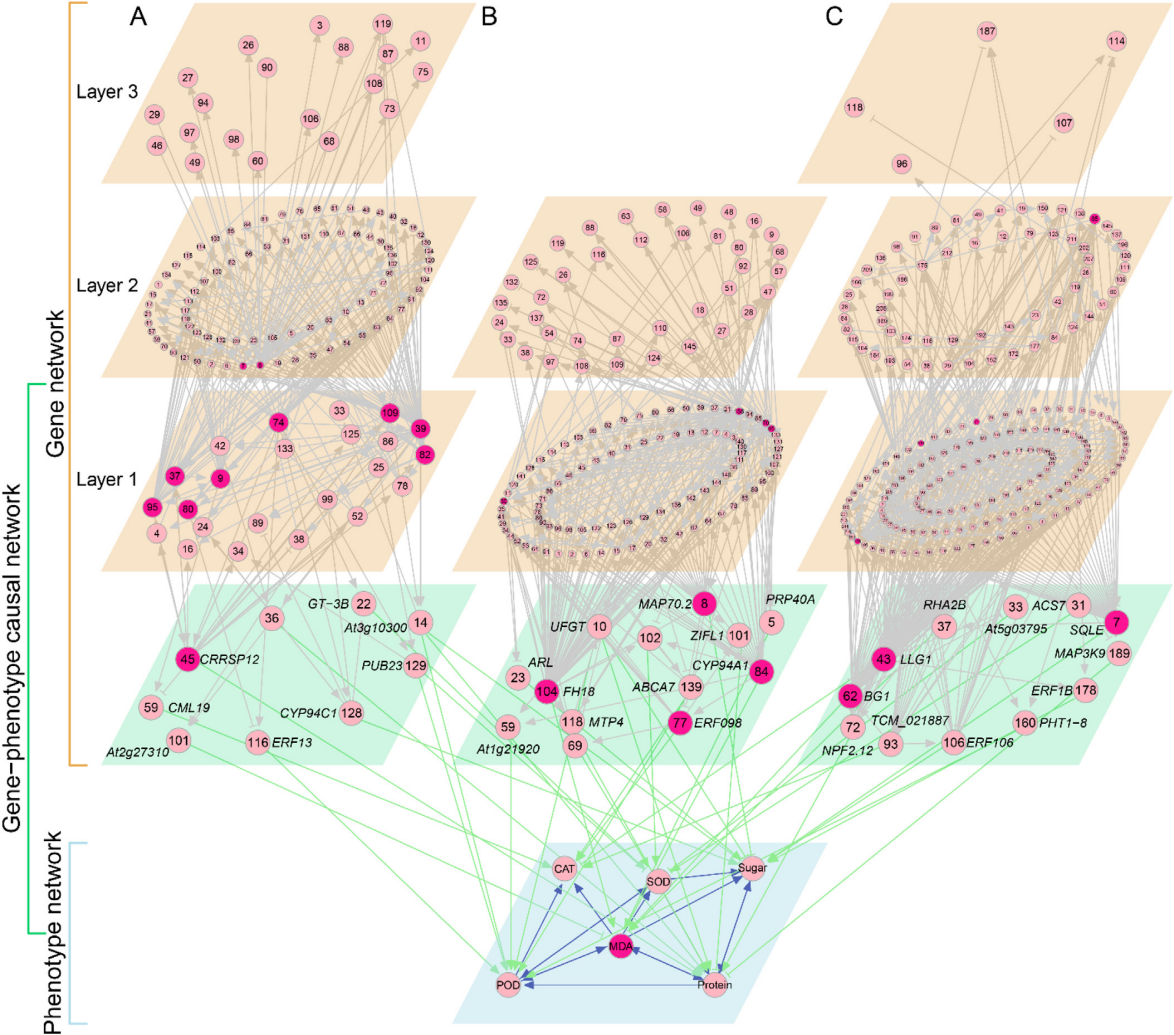
The regulatory networks among 136, 144 and 219 genes from modules 4 (A), 9 (B) and 10 (C), respectively, and their links to the phenotypic network of six physiological traits through causal networks. Hub genes within each module are named transcriptional factors that may be related to salt-resistant processes. Arrowed line denotes activation and T-shaped line indicates inhibition. The thickness of the lines stands for the strength of gene interaction. Some pairs of genes, like 4 and 9, are mutually regulated.

We reconstructed transcriptional networks among genes from three hub modules: 4 (136 genes), 9 (144 genes), and 10 (219 genes) (Fig. 4). In the network of module 4 (Fig. 4A), most gene regulations operate via directional synergism, accounting for 71.6% of the total number of links, suggesting that genes in this module tend to cooperate with other genes. This module contains seven hub genes that regulate numerous other genes. For example, genes 74 (*ERF061*) and 95 (*HLH92*) are transcriptional factors belonging to the EFR and BHLH transcriptional families, respectively, and are involved in response to salt stress in Arabidopsis [43], whereas gene 82 (*MAPK3*) recognizes and transfers the external stress signal. Genes 8 (*TCHQD*), 37 (*GWIN3*), and 109 (*EBF1*) are thought to interact with each other to regulate ethylene signals [44]. Many other genes can also be related to salt-tolerant processes in plants.

Module 9 contains several hub genes, such as genes 65 (*XTH3*), 70 (*GHAF1A*), 104 (*WAK2*) and 138 (not annotated) (Fig. 4B). These genes mediate biochemical processes related to the formation, growth and function of cell walls, although the exact mechanisms by which these genes play a role in salt stress resistance remain unknown. Module 10 has hub genes 7 (encoding *Squalene monooxygenase-like protein*), 43 (encoding *LORELEI-LIKE-GPI-ANCHORED PROTEIN 1*), 63 (*SLC50A1*), 85 (not annotated), 130 (*NDH2*) and 151 (not annotated) (Fig. 4C). These genes participate in regulating the transmembrane transportation of sugars [42].

Our model enables the visualization of how gene networks of each module determine the phenotypic network through a gene-phenotype causal network. The causal network contains genes that each have a direct link with the phenotypic network (downstream) and also, link with other genes in the upstream. The upstream genes that are situated peripherally to the phenotypic network may have an indirect impact on the traits. Based on their distances to the casual network, the upstream genes can be divided into a hierarchy (i.e., layer 1, layer 2, etc.). Results from different modules show that the causal network is composed of loosely linked genes, the majority of which are transcriptional factors characterized by a variety of distinct functions. In the casual network of module 4 (Fig. 4A), gene 45 is annotated as *CRRSP12*, which is involved in plant perception and response to biotic and/or abiotic stress signals [45]. Our result shows that *CRRSP12* plays a leadership role in regulating and organizing many other genes into a network, in addition to its direct effect on protein content (Fig. 4A), as has been observed in previous studies [45]. Gene 101 (*CML19*) within the causal network directly inhibits *MDA*, but the expression of MDA is strengthened by POD, which is activated jointly by gene 166 (*ERF-13*), 128 (*CYP94C1*), and 129 (*PUB23*), and by protein content, which is activated by gene 14 (*At3g10300*) and 36. Gene 36 is not annotated, suggesting a potential role that has not been detected previously. In the casual network of module 9 (Fig. 4B), gene 104, annotated as *FH18*, is a hub gene that directly links the hub trait MDA of the trait network. Because of its numerous links to other genes, its indirect impact on the trait network is also pronounced. The hub genes that play a similar role are also detected within the causal network of module 10 (Fig. 4C). Taken together, by reconstructing a cascade of hierarchic regulatory networks, our theory can precisely characterize how genes modulate the phenotypic plasticity of complex traits in response to any environmental change.

### 3.5 Methodology comparison and computer simulation

**Fig. 5:**
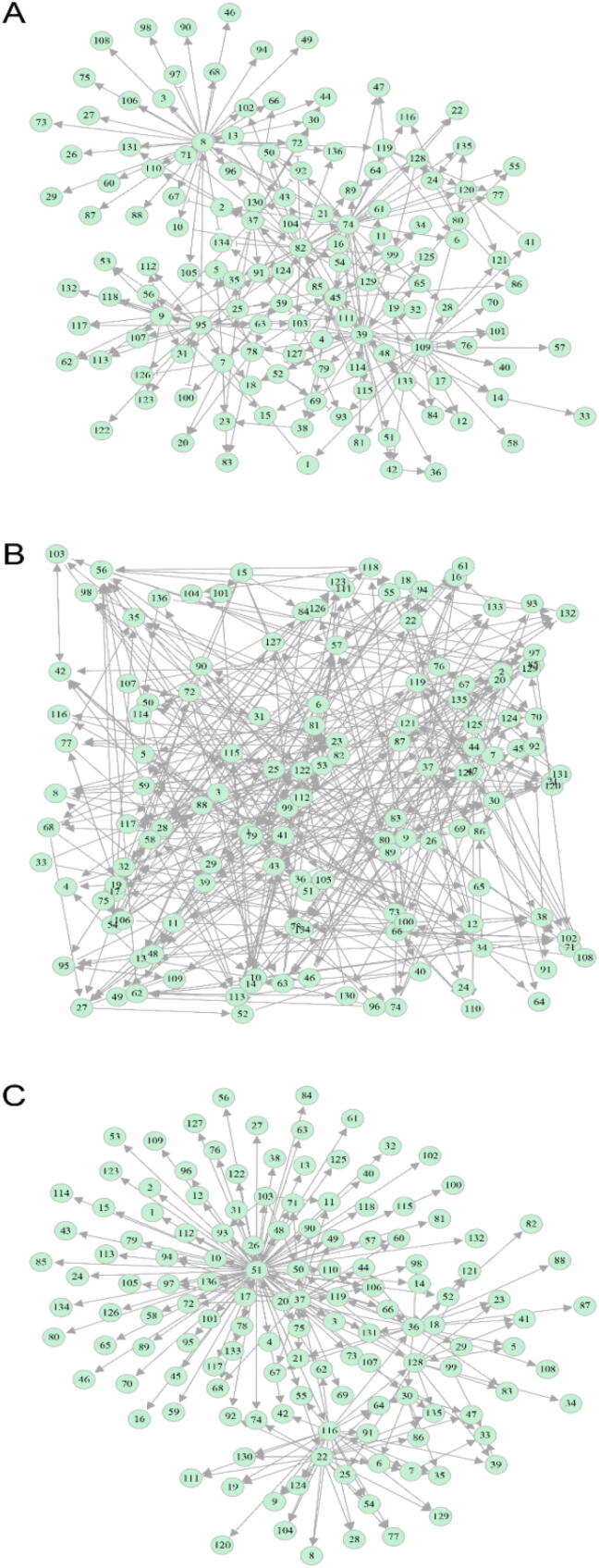
Gene regulatory networks among 136 genes from module 4 reconstructed by SEGM (A), G1DBN (B), and SA-ODE (C).

Dynamic Bayesian Networks (DBN) and ODE are two major approaches for reconstructing gene regulatory networks from temporal expression data. Lèbre [46] proposed a DBM method based on the concept of a low-order conditional dependence graph and implemented this method into an R package ‘G1DBN’. Wu et al. [47] proposed a sparse additive ODE (SA-ODE) method, coupled with variable selection, to construct dynamic gene networks. We use SEGN, G1DBN, and SA-ODE to simultaneously reconstruct a transcriptional network for module 4 (Fig. 5). Compared to SEGN (Fig. 5A), G1DBN produces a much denser network with poor sparsity in which no hub genes are detected (Fig. 5B). Both SEGN and SA-ODE identify many but different hub genes (Fig. 5A, 5C). As described above, hub genes detected by SEGN have biological meanings that are consistent with salt-resistant processes. However, hub genes by SA-ODE appears to be biologically less meaningful. For example, its hub gene 51 is a kunitz trypsin inhibitor, a protein that is synthesized when plants are subject to bacterial infection [48]. Although hub genes 36 and 37 (*GWIN3*) and 116 (*Putative ethylene-responsive transcription factor RAP2-13-like*) detected by SA-ODE are transcriptional factors, only 116 is found to be related with the salt stress response [49].

To compare the emergent properties of networks reconstructed from the three methods, we calculate six feature parameters. Connectivity is the number of nodes with which a node links within a network [38]; closeness describes the degree of linkage of one node to other genes [50]; betweenness reflects the importance of a node as a bridge across the network [51]; eccentricity is the longest distance of one node to other nodes [52]; eigenvector describes the importance of a node with respect to its neighboring nodes [53]; and PageRank evaluates the quality and quantity of links in a network [54]. Among the three methods, G1DBN performs worst, except for PageRank, with the connectivity of <10, closeness of <0.2, betweenness of <500, eccentricity of >10, and eigenvector of 0.01 (Table 3). These values explain the reason why G1DBN found no hub genes. We found that SEGN is better than SA-ODE in each of these criteria (Table 3).

**Table 3:** Average values of the centrality features for hub genes in the GRN of module 4 reconstructed by our SEGN model in a comparison between two existing approaches, G1DBN and GA-ODE.

Method	Connectivity	Closeness	Betweenness	Eccentricity	Eigenvector	PageRank
SEGN	14.947	0.407	812.115	3.842	0.157	0.008
G1DBN	6	0.156	202.103	11.158	0.015	0.007
SA-ODE	3.684	0.402	88.857	3.684	0.061	0.006

Note: Hub genes include genes 8, 39, 74, 82, 95 and 109 by GA-ODE.

**Fig. 6:**
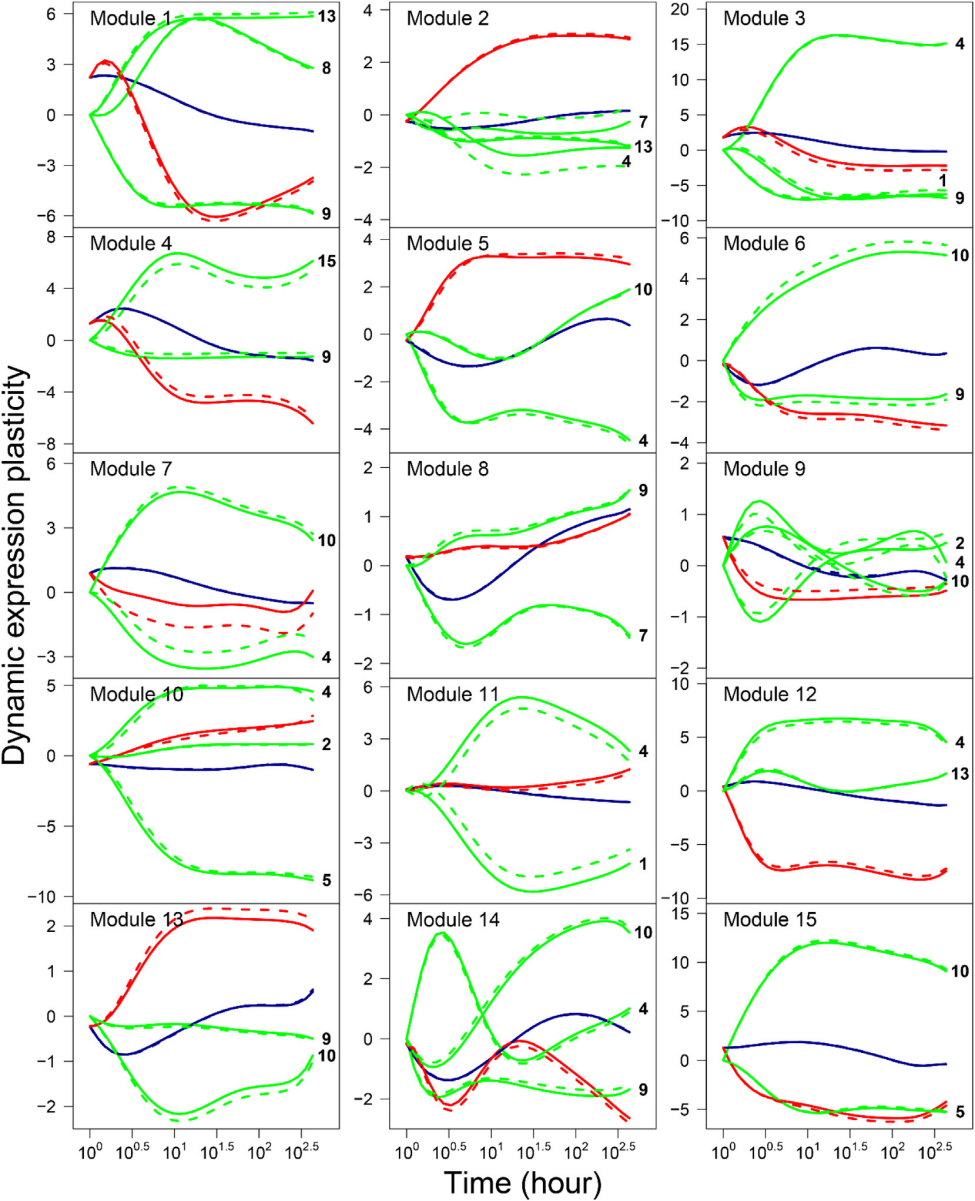
Estimated dynamic transcriptional plasticity (DTP) curves from our method, in a comparison with their underlying true curves. Solid red lines are the overall DTP curves, whereas broken and dotted lines are the independent and dependent DTP curves, respectively. Estimated and true curves are indicated in red and blue, respectively. (For interpretation of the references to colour in this figure legend, the reader is referred to the web version of this article.)

To validate the statistical advantage of SEGN, we simulated gene expression data under different scenarios and analyzed these data simultaneously with the new model, G1DBN and SA-ODE (Table S2). Fig. 6 illustrates the estimated independent and dependent DTP curves of all genes by the SEGN model, in comparison with their true curves under δ*_i_*^2^ = 0.01 and *T* = 30. We found that the estimated and true curves are broadly consistent, suggesting that our method has a good power for fitting and displays reasonably good statistical behavior for capturing the real patterns of gene-gene interactions within a gene network.

To compare the statistical efficacy of the different approaches, we calculated their true positives (TP), false positives (FP), true negatives (TN), false negatives (FN), true positive rates (TPR) expressed as TPR = TP/(TP + FN), and false positive rates (FPR) expressed as FPR = FP/(FP + TN). We also calculated the area under the curve (AUC) of the receiver operating characteristic curve (ROC) from the coordinates of TPR and FPR. Under the simulation scenario with δ*_i_*^2^ = 0.01 and *T* = 30, we found that SA-ODE performs better than G1DBH in many aspects, whereas SEGN is much better than SA-ODE in terms of every criterion (Table 4).

**Table 4:** Comparison of statistical properties of GRN reconstruction by our SEGN model, in a comparison with existing approaches, G1DBN and SA-ODE.

Method	TP	FP	TPR	FPR	AUC
G1DBN	6.9 (2.69)	53 (5.72)	0.08 (0.09)	0.06 (0.003)	0.52 (0.05)
SA-ODE	25.4 (2.38)	32 (3.68)	0.47 (0.09)	0.05 (0.003)	0.73 (0.02)
SEGN	35.2 (2.09)	10.3 (2.51)	0.78 (0.05)	0.01 (0.002)	0.88 (0.02)

## 4 Discussion

Although the study of gene regulatory networks as mediators of the response of phenotypic traits to environmental change is not new, this article, to our best knowledge, presents the very first computational model of its kind to reconstruct causal networks from genes to phenotypic plasticity. We integrate evolutionary game theory into a unified ODE framework, making it possible to infer systems evolutionary game networks (SEGNs). As biologically relevant networks, SEGNs can provide a quantitative characterization of bidirectional, signed, and weighted gene-gene interactions. The implementation of advanced statistical models, such as variable selection and functional clustering, equips SEGNs with the ability to handle the issue of high- or even ultrahigh dimensionality while preserving sparsity and omnidirectionality.

The most distinct feature of SEGNs may lie in their capacity to unravel real-time alterations of gene-gene interactions by which we can monitor how and when genes through their cooperation or competition drive an organism to best adapt to environmental change. By integrating phenotypic data, SEGNs can reconstruct causal links from gene interactions to phenotypic variation (see Fig. 4). From such hierarchical networks, we can identify (i) which genes directly affect a phenotype of interest, (ii) which genes indirectly affects this phenotype through their links with other genes, and (iii) which genes affect this phenotype by pleiotropically affecting other phenotypes that are correlated with the focal phenotype. SEGNs can characterize the magnitude and direction of these direct effects, indirect effects, and pleiotropic effects. With no doubt, these lines of information provide an unprecedented opportunity to understand the biological mechanisms underlying genotype-phenotype relationships and further design and engineer novel phenotypes through plant molecular design breeding [55].

We reconstructed SEGNs from salt-responding transcriptional data collected from *Populus euphratica*, in order to characterize how transcriptional factors communicate and coordinate with each other to determine network dynamics. Salt tolerance includes a complex web of interactive signals [40] and our SEGNs associated with salt tolerance can help geneticists to understand the mechanistic basis underlying how genes help plants limit the rate of salt uptake from the soil and the transport of salt throughout the plant, adjust the ionic and osmotic balance of cells in roots and shoots, and regulate leaf development and the onset of senescence [56], [57]. Based on the topological structure of SEGNs, we argue that salt-tolerant Euphrates poplars can be bred and selected more effectively by undersatnding and using genetic networks than by simply understanding individual functional genes.

Our motivation is to dissect the genetic networks of phenotypic plasticity for a desert woody plant in response to saline stress. However, the approach for network reconstruction is quite generic and, can be used to study the phenotypic plasticity of all other biological phenomena. For example, cancer cells display the ability to switch states or phenotypes in response to environmental fluctuations [58], [59]. The SEGNs of cancer-related phenotypic plasticity can help understand genetic signatures underlying this disease. Furthermore, recent developments in spatial and dynamic transcriptomic techniques have made it possible to probe the transcriptomes of single cells. SEGNs inferred from our model form a foundation for precise exploration of how genes interact with each other in cell-specific networks and how these networks cross-talk with biological or biomedical processes. The computational platform of SEGN reconstruction is flexible enough to be used on any kind of omics data, allowing other researchers to identify key interaction pathways by which genotype-phenotype relationships can be bettered mapped.

## 5 Data and code availability

The data and code uploaded at https://github.com/LiboJiang/EuphratesSEGN can be freely uploaded and used by researchers worldwide. They can also be requested from the corresponding author.

## CRediT authorship contribution statement

**Libo Jiang:** Formal analysis, Methodology. **Christopher H. Griffin:** Methodology. **Rongling Wu:** Conceptualization, Methodology, Project administration.

## Declaration of Competing Interest

The authors declare that they have no known competing financial interests or personal relationships that could have appeared to influence the work reported in this paper.
