# Supplementary material for: SEGN: Inferring real-time gene networks mediating phenotypic plasticity
Source: Comput Struct Biotechnol J. 2020 Sep 5;18:2510–21. doi: 10.1016/j.csbj.2020.08.029 (PMC7516210; doi:10.1016/j.csbj.2020.08.029)
Supplement: Supplementary data 1 [file mmc1.docx]

## Supplementary Text

## Statistical procedure for SEGN inference

## Likelihood

## Let y*_i_* = (*y_i_*(*t*_0_), *y_i_*(*t*_1_), …, *y_i_*(*t_T_*)) denote a vector of DTP values for gene *i* (*i* = 1, …, *m*) over all time points calculated by equation (2A). The likelihood function of model parameters φ = (μ, Σ) ∈ Φ given these data is written as

$\mathcal{L}$(**μ**, **Σ**) = *f*(??_1_, … , ??_??_: **μ**, **Σ**) (S1)

where *f*(⋅) is the *m*-variate *t_T_*-dimensional longitudinal multivariate normal distribution with mean

vector **g** = (**g**_1_, …, **g***_m_*) with **g***_i_* = (*g_i_*(*t*_0_), *g_i_*(*t*_1_), …, *g_i_*(*t_T_*)) and the covariance matrix of *e_i_*(*t*), **Σ**. As described below, we will model mean-covariance structures.

The time-varying mean values of DTP of each gene are modeled by a system of ODEs described by equation (3) containing the independent and dependent components. Each component is fitted by a nonparametric approach, such as B-splines, regression B-spline, penalized B-spline, local polynomials and Legendre orthogonal polynomials (LOP). Because of its advantage in orthogonality and efficient convergence, LOP has been used to model the curves of any complex form using sparse data in quantitative genetic studies (Das et al. 2011; Jiang et al. 2017). The LOP is a solution of the Legendre differential equation, (1 – *v*^2^)(*d*^2^*u*/*dv*^2^) – 2*v*(*du*/*dv*) + *r*(*r* + 1)*u* = 0. Let **P***_iR_*(*t*) = (*P_i_*_0_(*t*), *P_i_*_1_(*t*), …, *P_iR_*(*t*)) denote a vector of LOP including the first *R* orders for gene *i* at time *t*, and **α***_i_* = (*α_i_*_0_, *α_i_*_1_, …, *α_iR_*) denote a vector of basic values of time-invariant independent transcriptional plasticity of gene *i*. Then, the independent DTP of gene *i* is expressed as

$$g_{i}\left( t \right)=\mathbf{P}_{iR}^{T}\left( t \right)\boldsymbol{\alpha}_{i} (S2)$$

We took the DTP mean of three replicates as $g_{i}\left( t \right)$ to fit the above equation. An optimal order of LOP may be gene-specific; i.e., each gene may have a different LOP order of interaction by other genes. The optimal order for each gene *i* can be determined via an information criterion, such as AIC or BIC. The LOP-based transformation (S2) is used to fit the DTP of both independent and dependent expression as described in equation (3). Similarly, we use LOP to model time-varying dependent DTP components.

Since the residual covariance matrix **Σ** contains an autocorrelative structure, its structural modeling using a parsimonious time-series approach can increase the precision of ODE parameter estimation and computational efficiency. Given its power to structure the longitudinal covariance of quantitative traits in genetic mapping studies (Zhao et al. 2005a; Zhao et al. 2005b), the structured antedependence (SAD) model, developed by Zimmerman et al. (2001), is implemented into likelihood (4). The SAD assumes that residual errors at time t are not only composed of innovation errors specifically produced at this time point, but also contain a proportion of the residual errors from the preceding time points. The size of this proportion, i.e., the degree of antedependence (*ρ*), decays with time lag. The first-order SAD (SAD (1)) only considers the dependence of errors at the immediate time point. The innovation error for gene *i* is iid with mean zero and variance $\delta_{i}^{2}$which is assumed to be constant across time points. Two parameters, *ρ_i_* and $\delta_{i}^{2}$, can well model the structure of **Σ**.

With joint ODE and SAD modeling, the parameters involved in likelihood (S1) are re-written as **φ** = $\left\{ \Theta_{i},\Theta_{ii^{'}},\rho_{i},\delta_{i}^{2} \right\}_{i\neq i^{'}=1}^{m}$. We can obtain the optimal solution of these parameters by maximizing the likelihood (S1), expressed as

$$\hat{\boldsymbol{\phi}}\in\left\{ \arg\max_{\boldsymbol{\phi}\in\boldsymbol{\Phi}} \mathcal{L}\left( \boldsymbol{\mu}, \boldsymbol{\Sigma} \right) \right\}. \left( S3 \right)$$

We implement a hybrid algorithm of the fourth-order Runge-Kutta (RK4) algorithm and simplex approach to solve the likelihood incorporated by a system of LOP-transformed ODEs (equation (3)). Intuitively, this maximization that makes the data most probable implies an optimal topological structure and organization by which genes interact with each other to maximize the joint expression of all genes. This solution is therefore consistent with the basic principle of evolutionary game theory (Smith and Price 1973).

**Network sparsity**

ODEs in equation (3) represent a full model that systematically describes all possible interactions among the genes within a gene network. However, these interactions rarely occur at the same time in a single cell. Instead, a given gene may only interact with a certain subset of genes to form a sparse network. In modeling social networks, Dunbar (1992) found that there exists a limit for the number of relationships within a network an individual can stably maintain. This phenomenon, called Dunbar’s law, enlighten us to reconstruct a network based on some key pathways that form a network. Penalty-based variable selection has well been developed to choose a small set of predictors that are most significantly associated with a response.

By regressing the DTP of each gene *i* on the DTP of all other genes *i*′ (*i*′ = 1, …, *m*; *i*′ ≠ *i*) as predictors, we formulate a multiple regression model across time, where times behave like samples. A LASSO-based variable selection approach (Tibshirani 1996) can be implemented to shrink the dimension of links possibly owned by gene *i*. LASSO is particularly powerful for the penalty regression analysis of a response on an extremely large number of predictors across a much smaller size of samples. However, in a typical genomic experiment, we often regress on thousands of genes across only several or a dozen of time points. To resolve this curse of dimensionality, we interpolate DTP values as many as needed through a smoothing function. Genes change their expression in response to developmental cues, which has been found to obey some pattern. For example, if genes are periodically regulated, their time-dependent expression follow a periodic function (Dale et al. 2003). This provides a foundation for fitting time-varying changes of gene expression by a parametric or nonparametric approach (Kim et al. 2008). After fitting the time trend of gene expression under each treatment, we can potentially obtain an infinite number of DTP values over time based on equation (2A), which thus allows the number of samples to be matched with any number of genes. We implement group LASSO (Yuan and Lin 2006) and adaptive group LASSO (Wang and Leng 2008) to determine an optimal number of interacting genes (??_??_ ≪ ??) for each focal gene *i*.

## Network testing

## Since it is hardly possible that a gene is affected by all other genes simultaneously, we implement variable selection to choose a subset of significant genes (say *d_i_*) that affect a focal gene. In the end, we estimate all ODE parameters contained in equation (3). Next, we test whether or not the genes studied constitute a tight network. In so doing, we formulate a joint test of all dependent expression plasticity, expressed as

$$\left\{ \begin{matrix} H_{0}:\sum_{{i^{'}=1,i}^{'}\neq i}^{d_{i}} Q_{ii^{'}}\left( g_{i^{'}}\left( t:\Theta_{ii^{'}} \right) \right)=0 \\ H_{1}:\sum_{{i^{'}=1,i}^{'}\neq i}^{d_{i}} Q_{ii^{'}}\left( g_{i^{'}}\left( t:\Theta_{ii^{'}} \right) \right)\neq0 \end{matrix} (S4) \right.$$

for *i* = 1, …, *m*, where the likelihood under the H_0_ is formulated similarly to that of equation (S1). Thus, by calculating the likelihoods under the H_0_ and H_1_, we estimated the log-likelihood ratio (LR) as a test statistic used for the inference of accepting or rejecting the H_0_. If the null hypothesis is accepted, this implies that the network does not exist among these genes studied. We assume that the LR calculated from equation (S4) empirically follows a chi-square distribution with the degrees of freedom equal to the difference of the numbers of unknown parameters under the H_1_ and H_0_.

# Computer simulation

# We performed simulation studies to investigate the statistical behavior of the new model by perturbing conditions including residual errors and the number of time points (*T*). Assume that we obtain dynamic transcriptional plasticity (DTP) data of 15 genes, labeled from 1 to 15, across 10 and 30 time points, respectively. Each gene interacts only with a specific set of genes, which are specified by a system of ODEs as follows:

$\frac{dg_{1}}{dt}=Q_{1}\left( g_{1}\left( t:\Theta_{1} \right) \right)+Q_{1|8}\left( g_{8}\left( t:\Theta_{1|8} \right) \right)+Q_{1|9}\left( g_{9}\left( t:\Theta_{1|9} \right) \right)$+$Q_{1|13}\left( g_{13}\left( t:\Theta_{1|13} \right) \right)$

$\frac{dg_{2}}{dt}=Q_{2}\left( g_{2}\left( t:\Theta_{2} \right) \right)+Q_{2|4}\left( g_{4}\left( t:\Theta_{2|4} \right) \right)+Q_{2|7}\left( g_{7}\left( t:\Theta_{2|7} \right) \right)$+$Q_{2|13}\left( g_{13}\left( t:\Theta_{2|13} \right) \right)$

$\frac{dg_{3}}{dt}=Q_{3}\left( g_{3}\left( t:\Theta_{3} \right) \right)+Q_{3|1}\left( g_{3}\left( t:\Theta_{3|1} \right) \right)+Q_{3|4}\left( g_{4}\left( t:\Theta_{3|4} \right) \right)$+$Q_{3|9}\left( g_{9}\left( t:\Theta_{3|9} \right) \right)$

$\frac{dg_{4}}{dt}=Q_{4}\left( g_{4}\left( t:\Theta_{4} \right) \right)+Q_{4|9}\left( g_{9}\left( t:\Theta_{4|9} \right) \right)+Q_{4|15}\left( g_{15}\left( t:\Theta_{4|15} \right) \right)$

$\frac{dg_{5}}{dt}=Q_{5}\left( g_{5}\left( t:\Theta_{5} \right) \right)+Q_{5|4}\left( g_{4}\left( t:\Theta_{5|4} \right) \right)+Q_{5|10}\left( g_{10}\left( t:\Theta_{5|10} \right) \right)$

$\frac{dg_{6}}{dt}=Q_{6}\left( g_{6}\left( t:\Theta_{6} \right) \right)+Q_{6|9}\left( g_{9}\left( t:\Theta_{6|9} \right) \right)+Q_{6|10}\left( g_{10}\left( t:\Theta_{6|10} \right) \right)$

$\frac{dg_{7}}{dt}=Q_{7}\left( g_{7}\left( t:\Theta_{7} \right) \right)+Q_{7|4}\left( g_{4}\left( t:\Theta_{7|4} \right) \right)+Q_{7|10}\left( g_{10}\left( t:\Theta_{7|10} \right) \right)$

$\frac{dg_{8}}{dt}=Q_{8}\left( g_{8}\left( t:\Theta_{8} \right) \right)+Q_{8|7}\left( g_{7}\left( t:\Theta_{8|7} \right) \right)+Q_{8|9}\left( g_{9}\left( t:\Theta_{8|9} \right) \right)$

$\frac{dg_{9}}{dt}=Q_{9}\left( g_{9}\left( t:\Theta_{9} \right) \right)+Q_{9|2}\left( g_{2}\left( t:\Theta_{9|2} \right) \right)+Q_{9|4}\left( g_{4}\left( t:\Theta_{9|4} \right) \right)+Q_{9|10}\left( g_{10}\left( t:\Theta_{9|10} \right) \right)$

$\frac{dg_{10}}{dt}=Q_{10}\left( g_{10}\left( t:\Theta_{10} \right) \right)+Q_{10|2}\left( g_{2}\left( t:\Theta_{10|2} \right) \right)+Q_{10|4}\left( g_{4}\left( t:\Theta_{10|4} \right) \right)+Q_{10|5}\left( g_{5}\left( t:\Theta_{10|5} \right) \right)$

$\frac{dg_{11}}{dt}=Q_{11}\left( g_{11}\left( t:\Theta_{11} \right) \right)+Q_{11|1}\left( g_{1}\left( t:\Theta_{11|1} \right) \right)+Q_{11|4}\left( g_{4}\left( t:\Theta_{11|4} \right) \right)$

$\frac{dg_{12}}{dt}=Q_{12}\left( g_{12}\left( t:\Theta_{12} \right) \right)+Q_{12|4}\left( g_{4}\left( t:\Theta_{12|4} \right) \right)+Q_{12|13}\left( g_{13}\left( t:\Theta_{12|13} \right) \right)$

$\frac{dg_{13}}{dt}=Q_{13}\left( g_{13}\left( t:\Theta_{13} \right) \right)+Q_{13|9}\left( g_{9}\left( t:\Theta_{13|9} \right) \right)+Q_{13|10}\left( g_{10}\left( t:\Theta_{13|10} \right) \right)$

$\frac{dg_{14}}{dt}=Q_{14}\left( g_{14}\left( t:\Theta_{14} \right) \right)+Q_{14|4}\left( g_{4}\left( t:\Theta_{14|4} \right) \right)+Q_{14|9}\left( g_{9}\left( t:\Theta_{14|9} \right) \right)+Q_{14|10}\left( g_{10}\left( t:\Theta_{14|10} \right) \right)$

$\frac{dg_{15}}{dt}=Q_{15}\left( g_{15}\left( t:\Theta_{15} \right) \right)+Q_{15|5}\left( g_{5}\left( t:\Theta_{15|5} \right) \right)+Q_{15|10}\left( g_{10}\left( t:\Theta_{15|10} \right) \right)$

where *Q.*(⋅) and *Q.|.*(⋅) are the independent DTP of a given focal gene and its dependent DTP affected by other genes, respectively. We assume that the change of DTP is specified by LOPs of order 5 with basis values with assumed values given in Table S2. We further assume that residual errors follow a multivariate normal distribution with mean vector **0** and covariance matrix **Σ** that can be structured by SAD(1). The degree of antedependence is set as *ρ_i_* = 0.1 and innovative variance set as ??*_i_*^2^ =0.01 or 0.1 for gene *i* = 1, …, 15.

**Experimental design**

In 2011, we found an isolated wild stand of Euphrates poplar, composed of several thousands of mix-aged trees from young seedlings to saplings to senescent adult trees in an open land of salt soils in the central Inner Mongolia, China. Microsatellite marker analysis suggests that trees constituting this stand are derived from the same genotype. To study the mechanisms of this genotype to survive soil salinity, we sampled randomly 20 trees from the stand that are cloned through tissue culture into the same-aged juvenile trees grown in individual plots. These pots contain pearlite and vermiculite and were placed at a greenhouse under consistent temperature (22℃) and illumination conditions (16 h photo period) at Beijing Forestry University. The plants were watered with Hoagland nutrient solution irrigation every two weeks. After three months, the plants were about 50 cm in height. Twenty four uniformly growing plants were selected for the following salt stress treatment. They were randomly divided into two groups. One group was treated with 300mM NaCl solution, while the other group was treated with an equal amount of water and used as control. *P. euphratica* root samples were collected at 0 h, 1 h, 6 h, 24 h, 7 days and 18 days after the salt stress treatment; meanwhile the root samples in the control group were collected at 24 h and 7 days. At each time point, three plants were selected as biological replicates. After being collected, all the samples were immediately frozen in liquid nitrogen for RNA extraction and physiological measurements.

We measured six salt-responsive physiological traits from roots of Euphrates poplar clones, cultured in salt-free and salt-stress media, before treatment and at four different time points after treatment. The physiological traits measured include superoxide dismutase (SOD), malonaldehyde (MDA), catalase (CAT), peroxidase (POD), soluble sugar content, and protein content. Also, we used high-throughput RNA sequencing technique, the rRNA-depleted RNA by NEBNext® Ultra™ Directional RNA Library Prep Kit for Illumina® (NEB, USA), to obtain a total of 3,360,000,000 reads with error rate less than 0.02% from 24 samples of *P. euphratica* including the salt-treated samples and control samples. After remove the original reads with low quality, 3,200,000,000 sequencing reads were kept, leading to 479.12G clean data in total. Through aligning to the *P. euphratica* genome, we obtained a total of 344,341 transcripts, including 49,674 unigenes. These unigenes ranged from 300bp to 2000bp in length, with a mean of 909bp, indicating that it is reliable for transcriptome assembly. Among these unigenes, 1,819 were identified as salt-responsive genes using four different methods, including edgeR, DEseq2, limma-voom and maSigPro (Table S1). We will use these genes to reconstruct a salt-tolerant SEGN.

**References**

Das, K., Li, J., Wang, Z., Tong, C., Fu, G., Li, Y., Xu, M., Ahn, K., Mauger, D., Li, R., and Wu, R.L. (2011). A dynamic model for genome-wide association studies. *Hum. Genet.* 129(6): 629.

Dunbar, R.I.M. (1992). Neocortex size as a constraint on group size in primates. *J. Hum. Evol.* 22(6): 469-493.

Jiang, L., Zhang, M., Sang, M., Ye, M., and Wu, R.L. (2017). Evo-Devo-EpiR: a genome-wide search platform for epistatic control on the evolution of development. *Brief. Bioinform.* 18(5): 754-760,

Kim, B.R., Zhang, L., Berg, A., Fan, J., and Wu, R. (2008). A computational approach to the functional clustering of periodic gene-expression profiles. *Genetics* 180(2): 821-834.

Tibshirani, RJ. (1996). Regression shrinkage and selection via the LASSO. J. R. Stat. Soc. B. 58: 267-288.

Zhao, W., Chen, Y.Q., Casella, G., Cheverud, J.M., and Wu, R.L. (2005a). A non-stationary model for functional mapping of complex traits. *Bioinformatics* 21(10): 2469-2477.

Zhao, W., Hou, W., Littell, R.C., and Wu, R.L. (2005b). Structured antedependence models for functional mapping of multiple longitudinal traits. *Stat. Appl. Genet. Mol. Biol.* 4(1): 33.

Zimmerman, D.L., and Núñez-Antón, V. (2001). Parametric modelling of growth curve data: An overview. *Test* 10(1): 1-73.
