# Supplementary material for: SEGN: Inferring real-time gene networks mediating phenotypic plasticity
Source: Comput Struct Biotechnol J. 2020 Sep 5;18:2510–21. doi: 10.1016/j.csbj.2020.08.029 (PMC7516210; doi:10.1016/j.csbj.2020.08.029)
Supplement: Supplementary data 2 [file mmc2.docx]

## Supplementary Table S1 Hypothesized LOP parameters used to simulate the dynamic data of gene expression.

| **LOP** | $\beta_{1}$ | $\beta_{2}$ | $\beta_{3}$ | $\beta_{4}$ | $\beta_{5}$ | **LOP** | $\beta_{1}$ | $\beta_{2}$ | $\beta_{3}$ | $\beta_{4}$ | $\beta_{5}$ |
| --- | --- | --- | --- | --- | --- | --- | --- | --- | --- | --- | --- |
| **$f_{1}$** | -1.32 | 1.08 | 0.17 | -0.55 | 0.04 | $f_{9}$ | -0.23 | 0.16 | -0.07 | 0 | -0.19 |
| **$f_{1,8}$** | -1.54 | 1.38 | -0.43 | -0.49 | 0.22 | $f_{9,2}$ | -0.11 | 0.25 | -0.13 | -0.27 | 0.57 |
| **$f_{1,9}$** | -0.27 | 1.24 | -1.06 | 0.6 | 0.6 | $f_{9,4}$ | -0.17 | 0.11 | 0.14 | 0.05 | 0.16 |
| **$f_{1,13}$** | -0.39 | 0.54 | -0.74 | 0.15 | 0.09 | $f_{9,10}$ | 0.11 | -0.13 | -0.13 | 0.06 | -0.13 |
| **$f_{2}$** | -0.72 | 0.57 | -0.32 | -0.15 | -0.13 | $f_{10}$ | -0.45 | -0.07 | -0.36 | -0.02 | 0.07 |
| **$f_{2,4}$** | -0.09 | 0.21 | 0.01 | -0.08 | 0.03 | $f_{10,2}$ | -0.23 | 0.08 | -0.22 | -0.09 | 0.18 |
| **$f_{2,7}$** | -0.24 | 0.18 | 0.2 | -0.11 | -0.23 | $f_{10,4}$ | 0.08 | -0.1 | 0.38 | 0.11 | -0.04 |
| **$f_{2,13}$** | -0.15 | 0.01 | 0.1 | -0.28 | -0.05 | $f_{10,5}$ | 0.3 | -0.72 | 0.46 | -0.27 | -0.04 |
| **$f_{3}$** | -0.76 | 0.07 | -0.05 | -0.04 | 0.26 | $f_{11}$ | -0.4 | 0.2 | -0.02 | -0.47 | 0.05 |
| **$f_{3,1}$** | -0.71 | 0.53 | -0.13 | -0.16 | 0.18 | $f_{11,1}$ | -0.87 | 0.56 | 0.07 | -0.31 | -0.04 |
| **$f_{3,4}$** | 0.9 | -0.67 | 0.62 | 0.08 | -0.31 | $f_{11,4}$ | 0.81 | -0.32 | 0.07 | 0.28 | 0.02 |
| **$f_{3,9}$** | -1.01 | 1.61 | -1.19 | 0.41 | 0.42 | $f_{12}$ | -0.26 | -0.25 | -0.59 | 0.98 | -0.66 |
| **$f_{4}$** | 0.17 | 0.76 | -0.12 | -0.08 | 0.24 | $f_{12,4}$ | 0.27 | -0.33 | 0.01 | 0.11 | 0.44 |
| **$f_{4,9}$** | -0.14 | 0.4 | -0.14 | -0.03 | -0.08 | $f_{12,13}$ | 1.41 | -0.99 | -0.75 | 1.08 | 0.27 |
| **$f_{4,15}$** | -0.35 | 0.02 | 0.64 | -0.63 | -0.55 | $f_{13}$ | -0.23 | 0.34 | -0.5 | -0.36 | 0.02 |
| **$f_{5}$** | -0.5 | 0.05 | -0.15 | 0.34 | -0.21 | $f_{13,9}$ | 0.3 | -0.01 | 0.05 | 0.22 | 0.04 |
| **$f_{5,4}$** | 0.04 | -0.22 | -0.22 | 0.31 | 0.05 | $f_{13,10}$ | 0.22 | 0.28 | 0.48 | -0.37 | -0.3 |
| **$f_{5,10}$** | -1.1 | -0.25 | 0.79 | 0.28 | -0.2 | $f_{14}$ | -0.73 | 0.3 | 0.01 | -0.84 | 0.03 |
| **$f_{6}$** | -0.31 | -0.43 | 0.19 | -0.04 | 0.03 | $f_{14,4}$ | -0.21 | 0.7 | 0.05 | -0.59 | 0.12 |
| **$f_{6,9}$** | 0.07 | -0.02 | 0.12 | 0.06 | -0.67 | $f_{14,9}$ | 0.39 | -0.44 | 0.21 | 0.29 | -0.75 |
| **$f_{6,10}$** | -0.28 | 0.77 | 0.1 | 0.16 | -0.15 | $f_{14,10}$ | -0.49 | 0.39 | -0.1 | -0.19 | 0.39 |
| **$f_{7}$** | 1.42 | 0.72 | -0.91 | -1.24 | -0.57 | $f_{15}$ | -0.71 | -0.07 | -0.51 | -0.54 | -0.64 |
| **$f_{7,4}$** | -0.56 | -0.12 | -0.04 | 0.23 | 0.13 | $f_{15,5}$ | -0.12 | 0.08 | 1.11 | -0.1 | -0.17 |
| **$f_{7,10}$** | 0.09 | 0.16 | -0.61 | 0.47 | 0.17 | $f_{15,10}$ | 0 | 1 | -1.04 | 0.83 | 0.02 |
| $f_{8}$ | 0.25 | 0.07 | -0.14 | -0.01 | 0.03 |  |  |  |  |  |  |
| $f_{8,7}$ | 0.23 | -0.49 | -0.46 | 0.4 | 0.24 |  |  |  |  |  |  |
| $f_{8,9}$ | -0.83 | 0.02 | 0.56 | -0.35 | -0.46 |  |  |  |  |  |  |
